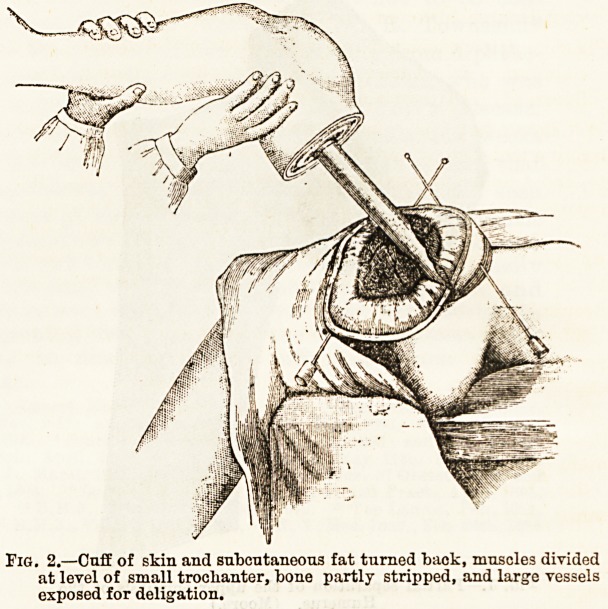# General Surgery

**Published:** 1894-07-28

**Authors:** 


### GENERAL SURGERY.

**Compound Fractures.**—Cases of compound fracture calling for amputation are exceedingly few. Gould[34] lays down an axiom that *there is no condition in a compound fracture which demands amputation which, if existing in a simple fracture, would not also demand primary amputation.* Primary amputation is only necessary when there is such interference with the circulation that the part below cannot be kept alive, or such injury to the soft parts that recovery with restoration of function is impossible. Asepsis is of greater importance than any other consideration. G. W. Crile[35] points out that laceration of great nerve trunks, though a serious complication, is not in itself a sufficient indication for amputation. All crushed and soiled portions of skin or soft parts should be removed and the normal relation of muscles and soft parts restored by means of buried sutures. In marked over-riding of fragments he employs ligatures of silk for direct fixation—binding the fragments together, in several places, like a bundle of sticks; the ligatures are cut off and buried in the wound. W. Fairbank[36] mentions an interesting compound fracture of the tibia and fibula in a healthy man, aged 47, caused by a sudden twist while playing cricket. He did not fall, but leant on his bat, and called out that his leg was broken. The case did well.

**Amputations**—Amputation at the hip joint by Wyeth's bloodless method has recently been improved upon by the author[37]. With the patient in the usual position for this form of amputation, the limb is emptied of blood in the usual way. While the member is elevated, or before the Esmarch's bandage is removed, the rubber tubing constriction is applied. The object of this constriction—and this is the chief point in the method—is the absolute occlusion of every vessel at the level of the hip joint safely above the field of operation; it permits the disarticulation to be completed and the vessels secured before the tourniquet is removed. Two large mattress needles or skewers, about three-sixteenths of an inch in diameter and ten inches long, are employed, one of which is introduced one inch below and slightly to the inner side of the anterior superior spine, and is made to traverse superficially the muscles and fascia on the outer side of the hip, emerging on a level with and about three inches from the point of entrance. The point of the second needle is made to enter one inch below the level of the crotch, internal to the saphenous opening, and passing squarely through the adductors, comes out an inch below the tuber ischii. The points are at once protected by bits of cork. A piece of strong white rubber tube, half-an-inch in diameter and long enough when tightened in position to go five or six times round the thigh, is now wound very tightly round and above the fixation needles and tied. Any of the methods of forming the flaps can now be carried out by the surgeon, and the vessels secured. Properly conducted, not a drop of blood should be lost, except that which was in the limb below the constriction when this was applied. If now the tourniquet be carefully and gradually loosened, each bleeding vessel may be secured as required, until the tube is entirely removed. W. H. Buechner[38] considers the principle of Wyeth's method so similar to that adopted by Volkmann, Veitch, and others, that the method cannot be properly so named. Professor Esmarch,[39] at the last meeting of German scientists and physicians, endeavoured to rehabilitate his bandage, making it the subject of his address. The objections to its use, he said, were the following. (1) The free hæmorrhage that took place after removal of the elastic bandage from paralysis of the vessels; (2) paralysis of nerves pressed on by the bandage; (3) death of flaps of skin, or margins of wounds; (4) the danger of entrance of septic material, &c., into the circulation. These objections constantly occurred through a perverted use of the bandage. Esmarch enumerated the precautions that had to be observed, and mentioned a number of other therapeutic measures —treatment of pseudo-arthroses and aneurisms, auto-transfusion, phlebostasis for the treatment of surgical tuberculosis, &c.—which had been practised with great success. On account of the frequent occurrence of gangrenous flaps in leg amputations, Bruns[40] has adopted a new subperiosteal method in twenty-two cases. A single circular incision is made, and the periosteum and over-

Fig. 1.—Needles and constrictor applied. Circular and longitudinal incisions for skin flap.

lying soft parts separated from the bones, which are then sawn through. If needed, to give better access to the depths of the wound, two lateral longitudinal incisions may be made. There was no instance of sloughing of the flaps, and the shape of the stump was excellent. In a recent article Geo. E. Marks[41] discusses the subject of amputation of the lower extremity hypothetically considered from a few standpoints: (1) Length of stump; (2) flaps; (3) disposition of cicatrices; (4) treatment of stumps after they have become healed; (5) time to apply an artificial leg. As to the last point, the artificial leg should be applied as soon as the stump is healed; this is especially important in the case of children, they are never too young to have the leg applied—it is the only means that will encourage heathful growth and symmetrical development. Torsion of arteries for the arrest of hæmorrhage is the subject of a paper by C. A. Dundore,[42] in which he gives a brief account of

Fig. 2.—Cuff of skin and subcutaneous fat turned back, muscles divided at level of small trochanter, bone partly stripped, and large vessels exposed for deligation.

the experience of some surgeons since Amussat's discovery and first observation in 1829. The method of its application has changed but slightly since this date. It is now well recognised to be less liable to be followed by secondary hæmorrhage; this has been absolutely proven by the experience in its use at Guy's Hospital. Murdoch has given some statistics of amputation in which torsion was used for the femoral, popliteal, axillary, tibial, brachial, radial, and ulnar arteries at the Western Pennsylvania Hospital, Pittsburg, with success in some hundreds of cases. After severe hæmorrhage, Cheron recommends[43] an artificial serum for hypodermic injection into the inner side of the thighs, the abdominal wall, or loose tissues of the back: Sulphate of sod. ʒii., phosphate of sod. ʒ; chloride of sod. gr. xxx., pure carbolic acid ♏xv., sterilized distilled water ʒxxvi.

[34] Clinical Journal, Jan. 17, 1894, p. 183. [35] Med. Record N. Y., Feb. 10, 1894, p. 169. [36] Lancet, March 17, 1894, p. 720. [37] N. Y. Medical Record, Jan. 13, 1894, and Medical News, vol. lxiii. No. 24. [38] N. Y. Medical Record, Jan. 27, 1894, p. 124. [39] Med. Record, Jan. 13, 1894, p. 64. [40] Beiträge zur Klin. Chirurg., bd x., Amer. J. Med. Sci., Nov., 1893, p. 605. [41] New York Med. Journal, Jan. 27, 1894. p. 113. [42] Int. Med. Mag., Jan., 1894. [43] Ther. Gaz., Feb. 15, 1894, and l'Union Médicale.

## Figures and Tables

**Fig. 1. f1:**
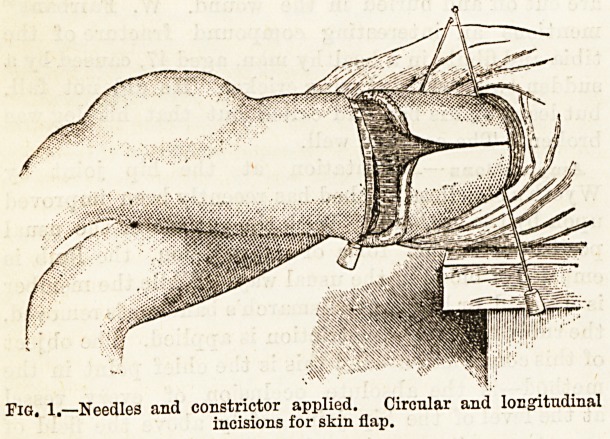


**Fig. 2. f2:**